# A Deeper Dive Into Advanced and Future Directions in Treating Patients With Acute Myeloid Leukemia

**Published:** 2018-04-01

**Authors:** Sandra E. Kurtin, Gabrielle Zecha

**Affiliations:** 1 The University of Arizona Cancer Center, Tucson, Arizona;; 2 University of Washington, Seattle, Washington

## Abstract

Acute myeloid leukemia is a disease that affects predominantly older patients, with a median age at diagnosis of 68. Overall prognosis is poor, but novel therapies that have emerged in recent years offer hope that outcomes can improve. In this comprehensive presentation, speakers covered a broad array of topics, including diagnostic workup, risk stratification, and novel therapeutic agents, and walked listeners through three illustrative case presentations.

Acute leukemia comprises a group of clonal neoplastic disorders of hematopoietic progenitor cells, one of the more common being acute myelogenous leukemia (AML). There were 21,380 new cases and 10,590 deaths AML-related deaths in 2017. The disease affects predominantly older patients with a median age at diagnosis of 68. The overall prognosis is poor, with a 5-year survival of about 27%. Multiple new and novel therapies have emerged in recent years, offering encouragement that outcomes in AML, including survival, will improve.

## RISK FACTORS, SIGNS, AND SYMPTOMS

A major contributing factor to the poor prognosis of AML is the lack of established risk factors that would afford opportunities for prevention. In more than 80% of new diagnoses, patients have no recognized risk factors, said Ms. Kurtin. Older age, male sex, and tobacco use (especially in older age) are possible risk factors. Certain environmental or occupational exposures have been implicated in the etiology of AML, along with iatrogenic and genetic factors that apply to a relatively small proportion of patients (Table 1).

**Table 1:**
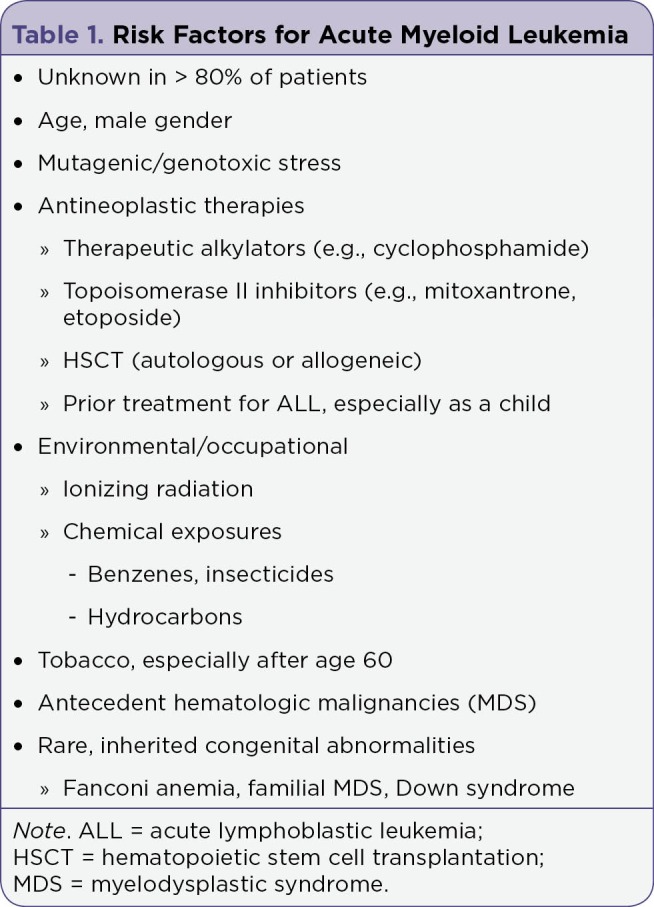
Risk Factors for Acute Myeloid Leukemia

Treatment-related AML (tAML) poses a unique challenge. There are two groups of patients with tAML; those whose disease occurs 5 to 10 years after treatment of the primary malignancy with regimens that include alkylating agents such as cyclophosphamide and other agents such as cisplatin and carboplatin, and those whose disease occurs 2 to 3 years after treatment of the primary malignancy with topoisomerase II inhibitors such as etoposide or the anthracyclines. High-dose chemotherapy used in stem cell transplants also contributes to the risk. "Knowing which treatments patients have received is an important factor in interpreting clinical data in these patients," noted Ms. Kurtin.

Signs and symptoms of AML include a number of nonspecific clues: fever, dyspnea, easy bruising, bleeding, petechiae, progressive fatigue and malaise, weight loss, and loss of appetite. In subtypes of AML, patients may develop skin nodules or gingival hyperplasia. Patients may present with active tumor lysis, active bleeding, or infection.

## DIAGNOSTIC EVALUATION

The diagnostic workup should focus on the onset of suspicious signs or symptoms, said Ms. Kurtin. Did the symptoms arise suddenly or gradually over time? Describing the symptom history can help establish the timing of disease onset and the tempo of the disease. In de novo AML, these generally appear abruptly, whereas in tAML or secondary AML (sAML), there may be a more gradual onset. 

Examination of medications and comorbidities may influence eventual treatment decisions for the patient with AML. Physical examination should be comprehensive to establish a baseline and identify any abnormal findings that might influence treatment decisions or require immediate attention (Kurtin, 2018).

A comprehensive laboratory analysis is essential to evaluate major organ function and the characteristics of the disease (Dohner et al., 2017; Kurtin, 2018). Human leukocyte antigen is needed in patients eligible for transplant. A lumbar puncture with analysis of the cerebrospinal fluid is recommended. Testing for HIV and hepatitis is advisable because those conditions increase the risk of treatment-related morbidity. A pregnancy test is indicated for female patients of childbearing age.

A bone marrow biopsy is critical to the diagnostic workup (Döhner et al., 2017; Kurtin, 2018). The sample should be sufficient to adequately conduct testing required to make a diagnosis. Cytogenetics, fluorescence in situ hybridization (FISH) and flow cytometry are standard (Döhner et al., 2017). Testing for genetic mutations should include *NPM1*, *CEBPA*, *RUNX1*, *FLT3*, *TP53*, *ASXL1*, and *IDH2*. Gene rearrangements associated with AML include *PML-RARA*, *CBFB-MYH11*, *RUNX1-RUNX1T1*, *BCR-ABL1*, and other fusion genes.

With respect to diagnostic imaging, a baseline chest x-ray is indicated for all patients, said Ms. Kurtin. Because certain therapies for AML are cardiotoxic—particularly anthracyclines—a baseline cardiac assessment is advisable—electrocardiogram, multigated acquisition scan, or echocardiogram. If central nervous system involvement or cerebrovascular hemorrhage is a potential concern, computed tomography imaging is indicated. If leukemia meningitis is a potential concern, the patient should have a brain magnetic resonance imaging.

"If you’re in doubt, call radiology and say, ’Here’s what I’m worried about,’ " said Ms. Kurtin. "They’re going to say, ’Here’s what you want to order.’ So, we’re not wasting time or money or unnecessarily exposing patients to contrast dye and radiation."

## DIAGNOSTIC CLASSIFICATION

The capability to associate genetic mutations to specific subtypes of leukemia has provided the basis for more specific, or personalized risk stratification, and in some cases treatment selection. At the same time, defining the disease has become more complicated.

In the 2016 World Health Organization (WHO) classification of myeloid neoplasms, AML is characterized as a complex, dynamic disease with multiple somatically acquired driver mutations, coexisting competing clones, and disease evolution over time (Arber et al., 2016). There are multiple categories based on the predominate genetic signature.

The classification system distinguishes AML with recurrent genetic abnormalities, AML with myelodysplasia-related changes, therapy-related myeloid neoplasms, and AML not otherwise specified.

"We’re basically carving out niches for these diseases," said Ms. Kurtin. "We don’t completely understand all of these niches, but it’s becoming more and more clear."

## RISK STRATIFICATION

Improved understanding of the disease process has facilitated the development of risk strata for patients with AML, which guides decision-making about treatment, particularly the aggressiveness of treatment (Arber et al., 2016). Factors associated with poor risk are older physiologic age, poor performance status, and complex or poorly controlled comorbidities. Such patients are not considered candidates for intensive therapy, said Ms. Kurtin.

AML genetics also guide treatment decisions (Döhner et al., 2017). Patients with translocation (8;21)(q22;q22.1), for example, have a favorable prognosis. Mutant *NMP1* and *FLT3-ITD*^High^ are genetic alterations associated with intermediate risk. Multiple genetic factors are associated with adverse risk, including mutant t(6;9)(p23;q34.1) and nonmutant (wild-type *NPM1*) genetic factors.

"Most people will not be cured without an allogeneic stem cell transplant," said Ms. Kurtin. "Autologous transplants are not effective for myeloid malignancies. Allogeneic transplants are required to overcome the malignant clone."

**Figure 1:**
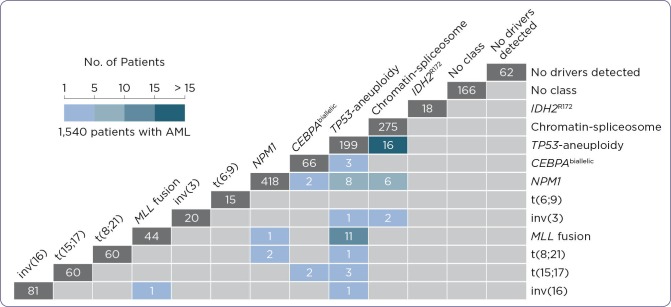
Predominant driver mutations in acute myeloid leukemia. AML – acute myeloid leukemia. Adapted from Papaemmanuil et al. (2016)

Identification of predominant driver mutations in AML has created potential for targeted treatment of the disease (Papaemmanuil et al., 2016). Additionally, creating a profile that reflects disease genetics across the spectrum from favorable to unfavorable can further inform treatment decisions (Figure 1).

*TP53* mutation and 17p deletion inherently confer a poor prognosis, which can improve or worsen when other genetic factors enter the profile. For example, a complex karyotype (defined as more than three abnormalities on metaphase) combined with a *TP53* mutation represents an especially unfavorable genetic profile for AML.

Treatment-related AML, arising from treatment of a different malignancy, and sAML, arising from an antecedent hematologic malignancy, most often myelodysplastic syndromes (MDS), are associated with an unfavorable prognosis, said Ms. Kurtin. Multiple potential mutations are associated with each condition, and the specific genetic profile associated with the disease influences the prognosis.

## TREATMENT APPROACH

In most cases, treatment begins as soon as the diagnosis and disease characteristics are confirmed. However, tests to confirm the diagnosis, including cytogenetic and molecular testing, may require a week or more if specimens must be sent to outside laboratories for processing. "In older patients where there is a suspicion of sAML or in many patients with tAML, studies have shown that waiting for these results to guide treatment decisions will not affect overall outcomes. However, this may not be true for de novo AML or in patients with aggressive disease associated with secondary systemic processes."

The key question is whether a patient is a candidate for transplantation. Allogeneic bone marrow transplantation is currently the only treatment that offers the potential to cure AML. Suitability for transplantation determines the intensity of all other therapy, said Ms. Kurtin.

The criteria for determining transplant eligibility have evolved considerably in recent years (Sorror et al., 2005, 2014, 2017). The Hematopoietic Cell Transplantation–Comorbidity Index (HCT-CI) comprises a combination of disease attributes, comorbid conditions, organ function, and age. An HCT-CI score greater than 3 is associated with inferior outcomes (Sorror et al., 2014). Starting with a score of 1, incremental increases in the HCT-CI for AML have a near-linear association with decrements in survival.

For patients who are medically fit for allogeneic transplantation, the process begins with the long-time standard for induction of 7 + 3: 7 days of cytarabine followed by 3 days of an anthracycline (O’Donnell et al., 2017). If the marrow is clear at day 14, the patient receives supportive care until bone marrow recovery. If the bone marrow remains clear at day 28, consolidation therapy can begin. If day-14 bone marrow assessment shows residual disease, the patient receives a second course of induction therapy. If disease persists at day 28, options for salvage therapy should be considered, or the patient might enroll in a clinical trial. People have experimented with the timing and mix of drugs, but the basic regimen has changed little since it was introduced in 1973.

If a patient is medically unfit for intensive induction therapy, treatment decisions (including intensity) will be guided by the trajectory of the disease, defined by cytogenetics and risk factors (Brandwein et al., 2017; O’Donnell et al., 2017). The patient should also be considered for enrollment in a clinical trial.

## NOVEL THERAPEUTIC AGENTS

Several novel therapies have emerged recently in the search for safer and more effective treatment of AML. The evidence reported to date reflects varying degrees of success in reaching the goal (Table 2).

**Table 2:**
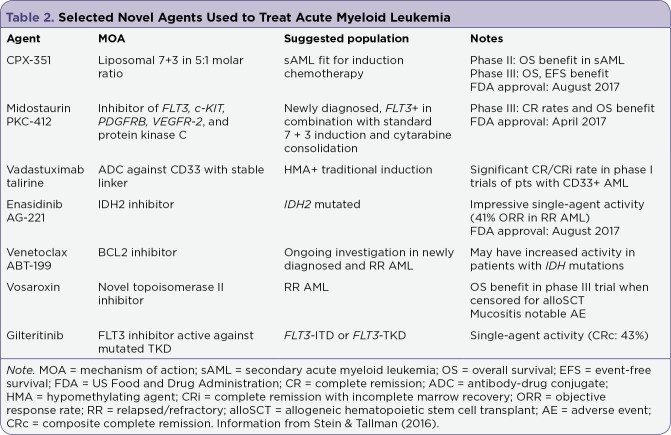
Selected Novel Agents Used to Treat Acute Myeloid Leukemia

**CPX-351**

This liposomal formulation of daunorubicin and cytarabine migrates into the bone marrow, where drug-filled liposomes are taken up to a greater extent by leukemic vs. normal marrow cells. Degradation of the liposomes releases the drug cargo into the intracellular environment (Kim, Gerhard, Harasym, Mayer, & Hogge, 2011). In a randomized trial involving high-risk older patients with AML, treatment with CPX-351 (Vyxeos) led to improved overall survival and lower mortality at 30 and 60 days, as compared with the traditional 7 + 3 regimen (Lancet et al., 2017).

"There was a greater depth of response and more complete responses [with CPX-351]," said Ms. Kurtin. "We have to start thinking in a different way and move away from the standard paradigm of day 14 and day 28, because we may not be able to retreat patients until week 6 or 8. This may create a sense of concern because we’re so used to the 14 and 28 paradigm that we’ve been doing for 44 years. We have to rethink our paradigm with a lot of these new drugs."

**Midostaurin**

This drug targets *FLT3*, a cell surface tyrosine kinase commonly mutated in leukemia and associated with poor prognosis (Griffith et al., 2004). The drug was evaluated as add-on therapy to the 7 + 3 regimen in a phase III randomized trial of patients with AML (Stone et al., 2017). The primary results showed a 23% reduction in the risk of death in patients who received midostaurin (Rydapt) in addition to daunorubicin and cytarabine. Grade ≥ 3 anemia and rash occurred more often with midostaurin, but otherwise, the safety profile was similar between the two treatment arms.

**Gilteritinib (ASP2215)**

Another *FLT3* inhibitor, this drug has activity against the *FLT3*-ITD activating mutation and the *FLT3*-D385 resistance mutation. In a single-arm study involving mostly patients with *FLT3*+ AML, a gilteritinib dose ≥ 80 mg resulted in a median overall survival of 31 weeks and a median duration of response of 20 weeks (Perl et al., 2016). A phase III trial involving patients with relapsed/refractory *FLT3*+ AML is underway.

**Enasidenib (AG-221)**

The first drug that specifically targets mutant *IDH2*, enasidenib (Idhifa) induces bone marrow differentiation and maturation as opposed to ablating leukemic cells. In a phase I/II trial, complete response with or without complete hematologic recovery occurred in 28% of patients with relapsed/refractory, *IDH2*+ AML (Stein et al., 2017).

Because of its unique mechanism of action, treatment with enasidenib initially may result in an increase in leukemic cells, followed by a dramatic decrease, said Ms. Kurtin. Several weeks of treatment may be required before response is apparent.

"This is another paradigm shift," she said. "We’re used to seeing all those numbers go down and bottom out. When that doesn’t happen, and it’s AML, we get a little uncertain. You have to understand the mechanism of action of this drug."

"You’re changing the dynamics of the disease with this drug, and that’s going to take a little bit of time," she added.

**Gemtuzumab Ozogamicin**

Initially approved by the US Food and Drug Administration in 2000, the anti-CD33 antibody-drug conjugate did not demonstrate improvement in remission rates in five clinical trials that evaluated the addition of gemtuzumab ozogamicin (Mylotarg) to standard induction therapy. However, the relapse rate was reduced in four of the trials, and two demonstrated a significant survival benefit. The drug was withdrawn from the US market in 2010 and then reintroduced at a lower dose in September 2017, following a reanalysis of the completed clinical trials (Hills et al., 2014).

**Vosaroxin**

A first-in-class anticancer quinolone derivative, vosaroxin intercalates DNA and inhibits topoisomerase II, leading to replication-dependent, site-selective DNA damage, G2 arrest, and apoptosis. A randomized trial showed no improvement in overall survival when vosaroxin was added to cytarabine for the treatment of relapsed/refractory AML. However, a subset analysis that excluded patients who underwent transplantation yielded a modest but statistically significant survival benefit (Sayar & Bashardoust, 2017).

**Venetoclax**

This drug specifically targets *BCL-2*, which plays a critical role in mitochondrial-mediated apoptosis and is overexpressed in AML. In a phase II trial of patients with high-risk, relapsed/refractory AML or who were unfit for chemotherapy, venetoclax (Venclexta) resulted in an overall response rate of 19%. Common adverse events included nausea, vomiting, febrile neutropenia, and hypokalemia (Konopleva et al., 2016).

Venetoclax was also evaluated in a phase Ib trial of combination therapy with a hypomethylating agent for older patients (median age 73) with newly diagnosed AML. The combination resulted in a complete response rate (with or without complete hematologic recovery) of 71% in 34 patients (DiNardo et al., 2015). Ongoing studies of venetoclax-containing combinations are ongoing, but preliminary data demonstrated significant improvement in response rates, particularly in older patients and those with high-risk disease attributes, said Ms. Kurtin.

## CASE PRESENTATIONS

The following case presentations illustrate how new and novel therapies for AML are being used in clinical practice to address specific patient and disease characteristics and to improve outcomes.

**Case 1: A 36-year-old man with newly diagnosed AML**

This 36-year-old man presented with a high white blood cell (WBC) count of 84,000/μL, mild anemia reflected in a hemoglobin of 14 g/dL, a platelet count of 131,000/μL, and 30% circulating blasts. Analysis of mutation status showed that he was *NPM1*- and *IDH2*-positive but *FLT3*-negative. Cytogenetic analysis revealed deletion 16q.

Echocardiography showed he had a normal level ejection fraction of 65%, and his physical exam was otherwise unremarkable, said Ms. Zecha.

The patient started induction therapy with granulocyte colony-stimulating factor plus the combination of cladribine, cytarabine, and mitoxantrone (G-CLAM). A bone marrow evaluation showed persistence of minimal residual disease, and the patient had another round of G-CLAM induction, followed by consolidation with G-CLA.

Several notable toxicities occurred during the course of treatment. As expected, the patient developed pancytopenia and became transfusion dependent. He also had significant mucositis and developed neutropenic fever. Subsequently, the patient became bacteremic, and blood cultures revealed the presence of multidrug resistant *Escherichia coli*. He became septic, which led to a stay in the intensive care unit (ICU), where he immediately received evidence-based treatment for sepsis, said Ms. Zecha.

Once he recovered from his ICU stay and his counts had normalized, a bone marrow biopsy was obtained and showed normal morphology, he was no longer *NPM1*-positive, and his cytogenetic abnormality had resolved.

The patient underwent consolidation treatment with high-dose cytarabine (HiDAC), but he presented with circulating blasts on day 22. He did not respond to treatment with cytarabine and decitabine, indicative of relapsed/refractory disease. Subsequently, treatment was initiated with intermediate-dose cytarabine and enasidenib that caused substantial nausea, which was managed with ondansetron.

"He actually did really well on this therapy," said Ms. Zecha.

**Case 2: A 72-year-old man with progressive fatigue**

The patient was a 72-year-old man who presented to the emergency department with fever, flu-like symptoms, and profound fatigue that had been present for quite a while, but had recently gotten significantly worse. His laboratory findings were notable for a slightly elevated serum creatinine (1.35 mg/dL), markedly elevated lactate dehydrogenase (LDH; 629 U/L), and decreased albumin of 3.1 g/dL. He had an international normalized ratio (INR) of 1.26. Other findings included a WBC count of 161,000/μL, hemoglobin of 6.8 g/dL (anemic), platelet count of 13,000/μL (thrombocytopenic), and 89% circulating blasts.

The patient was admitted to the hospital and received intravenous antibiotics. He received hydroxyurea and fluids until the workup was completed, said Ms. Zecha.

A review of the patient’s medical history showed that he had Hodgkin lymphoma at age 51, for which he received standard treatment (doxorubicin [Adriamycin], bleomycin, vinblastine [Velbe], and dacarbazine [ABVD] chemotherapy and radiation therapy). He also had a history of hypertension and hypercholesterolemia. His echocardiography revealed a left ventricular ejection fraction of 52%. The patient had no regular medical provider for the previous 16 years.

The elevated WBC and LDH, along with increased serum creatinine, increased his risk of tumor lysis syndrome, Ms. Zecha noted. The patient’s presentation, age, laboratory evidence, and clinical factors pointed to AML.

The patient’s bone marrow showed 90% myeloid blasts, *NPM1*-positivity, and *FLT3*-negativity. Cytogenetic analysis showed del(5q) and +8 (suggestive of AML preceded by a myelodysplastic syndrome, possibly precipitated by the treatment for Hodgkin lymphoma).

Older patients with AML and comorbid conditions or other risk factors have a poor prognosis and a high risk of treatment-related mortality. With a standard approach to therapy, he would have a treatment-related mortality of 90% (Walter et al., 2011).

"We’re trying to think outside the box about what we could do for this gentleman," said Ms. Zecha. "He was enrolled in a trial of CPX-351. He got induction therapy in an outpatient setting—mostly because he refused to be admitted—and he did really well. His son came up from California and was his primary caregiver, and he was coming back to our center 3 to 5 days a week." The drug can result in prolonged episodes of cytopenias, and the patient did experience that to some extent, but not to the point of interfering with treatment.

The patient’s 28-day bone marrow assessment showed persistent disease with 40% blasts. He had reinduction with CPX-351, which he tolerated well for the most part. He did require admission for neutropenic fever and was transfusion dependent. By day 36, his counts had recovered and a marrow was obtained, which showed no morphologic evidence of AML, although he remained *NPM1*+.

The patient went on to complete two cycles of consolidation with CPX-351, then was lost to follow-up. He subsequently returned 1 year later after calling to say, "I can’t even get out of bed." He had relapsed disease associated with 42% blasts. Nonetheless, the patient had an excellent response that allowed him to live a year, even though he began with a risk profile associated with a prognosis measured in days or weeks, said Ms. Zecha.

**Case 3: A 62-year-old woman with a 3-year history of thrombocytopenia that progressed to pancytopenia**

At presentation, this 62-year-old woman had a WBC of 49,000/μL, Hgb of 12.2 g/dL, platelet count of 45,000/µL, and circulating blasts of 76%. The patient had a normal chemistry panel, including serum creatinine of 0.8 mg/dL, LDH of 224 U/L, and uric acid of 4.8 mg/dL. Analysis of bone marrow aspirate showed 60% blasts, and cytogenetic abnormalities of trisomy 8, *NPM1*-positivity, and *FLT3*-positivity. Aside from heightened anxiety, the patient’s physical and clinical exam were unremarkable, said Ms. Zecha.

The patient started induction therapy with the standard 7 + 3 regimen, administered on an inpatient basis. No infusion-related toxicities or cerebellar disturbances occurred, and the patient was discharged on day 6, following accepted criteria for early discharge. She started induction with midostaurin at 50 mg po twice daily on days 8 to 21.

The midostaurin was generally well tolerated, although the patient remained pancytopenic and transfusion dependent. She also had nausea and mild mucositis. An episode of facial cellulitis required readmission on day 15, but she was discharged after a course of intravenous antibiotics.

The patient’s day 28 bone marrow evaluation revealed no evidence of AML, so she received HiDAC consolidation treatment, followed by early discharge and then midostaurin consolidation on days 8 through 21. No unusual or unexpected toxicities arose; she experienced nausea, anorexia, neutropenic fever, pancytopenia, and transfusion dependence.

The patient subsequently presented to the bone marrow transplant service in first complete remission for a planned matched allogeneic peripheral blood stem cell transplant.

"These cases illustrate the many tools we have available to treat our patients; they can help direct your therapy and result in better outcomes for your patients," said Ms. Zecha.
